# Alendronate prevents glucocorticoid-induced osteoporosis in patients with rheumatic diseases: A meta-analysis

**DOI:** 10.1097/MD.0000000000003990

**Published:** 2016-06-24

**Authors:** Shun-Li Kan, Zhi-Fang Yuan, Yan Li, Jie Ai, Hong Xu, Jing-Cheng Sun, Shi-Qing Feng

**Affiliations:** a Department of Orthopedics, Tianjin Medical University General Hospital; b School of Nursing, Tianjin Medical University, Heping, Tianjin, China.

**Keywords:** alendronate, glucocorticoid-induced osteoporosis, meta-analysis, rheumatic diseases

## Abstract

Glucocorticoid-induced osteoporosis (GIOP) is a serious problem for patients with rheumatic diseases requiring long-term glucocorticoid treatment. Alendronate, a bisphosphonate, has been recommended in the prevention of GIOP. However, the efficacy and safety of alendronate in preventing GIOP remains controversial. We performed a meta-analysis to investigate the efficacy and safety of alendronate in preventing GIOP in patients with rheumatic diseases.

We retrieved randomized controlled trials from PubMed, EMBASE, and the Cochrane Library. Two reviewers extracted the data and evaluated the risk of bias and quality of the evidence. We calculated the risk ratio (RR) with a 95% confidence interval (CI) for dichotomous outcomes, and the mean difference (MD) with a 95% CI for continuous outcomes using Review Manager, version 5.3.

A total of 339 studies were found, and 9 studies (1134 patients) were included. Alendronate was not able to reduce the incidence of vertebral fractures (RR = 0.63, 95% CI: 0.10–4.04, *P* = 0.62) and nonvertebral fractures (RR = 0.40, 95% CI: 0.15–1.12, *P* = 0.08). Alendronate significantly increased the percent change in bone mineral density (BMD) at the lumbar spine (MD = 3.66, 95% CI: 2.58–4.74, *P* < 0.05), total hip (MD = 2.08, 95% CI: 0.41–3.74, *P* < 0.05), and trochanter (MD = 1.68, 95% CI: 0.75–2.61, *P* < 0.05). Significant differences were not observed in the percent change in BMD at the femoral neck (MD = −0.33, 95% CI: −2.79 to 2.13, *P* = 0.79) and total body (MD = 0.64, 95% CI: −0.06 to 1.34, *P* = 0.07). No significant differences in the adverse events were observed in patients treated with alendronate versus the controls (RR = 1.00, 95% CI: 0.94–1.07, *P* = 0.89). The odds of gastrointestinal adverse events were significantly reduced (RR = 0.77, 95% CI: 0.62–0.97, *P* < 0.05).

Our analysis suggests that alendronate can increase the percent change in BMD at the lumbar spine, total hip, and trochanter, and is not associated with an increased incidence of gastrointestinal adverse events; however, the vertebral and nonvertebral fractures cannot be reduced. However, the results should be interpreted with caution due to the poor statistical power.

## Abstract 2

Supplemental Digital Content is available in the text

## 1 Introduction

Rheumatic diseases may cause significant swelling and pain in the joints and muscles, and ultimately result in a reduced quality of life. Glucocorticoids are frequently used as an immunosuppressive agent in rheumatic diseases.^[1]^ Glucocorticoids may well improve rheumatic symptoms and delay disease development. However, glucocorticoid-induced osteoporosis (GIOP) is a serious problem for patients with rheumatic diseases requiring long-term glucocorticoids treatment,^[2]^ and ultimately results in fractures in 30% to 50% of patients.^[3,4]^ Thus, the early prevention of GIOP is significantly important when glucocorticoids are used to manage rheumatic diseases.

Bisphosphonates have been shown to be a potent therapy for GIOP, and increase the bone mineral density (BMD) in patients receiving glucocorticoid treatment.^[5]^ Alendronate, a bisphosphonate, has been recommended for use in preventing GIOP.^[6]^ However, the efficacy and safety of alendronate in preventing GIOP in patients with rheumatic diseases remains controversial.

A recent trial^[7]^ demonstrated that alendronate significantly reduced the risk of vertebral fractures in patients with rheumatic diseases. On the other hand, no statistically significant differences in the incidence of vertebral fractures were found in another trial^[8]^ or a recent meta-analysis.^[9]^ However, this meta-analysis emphasized the use of alendronate in preventing and treating GIOP in patients with rheumatic diseases rather than primary prophylaxis, and did not evaluate the risk of bias and the quality of the evidence for each outcome. Therefore, the efficacy and safety of alendronate in preventing GIOP in patients with rheumatic diseases is still debated. We aimed to conduct a meta-analysis of randomized controlled trials to evaluate the efficacy and safety of alendronate in preventing GIOP in patients with rheumatic diseases.

## 2 Materials and methods

### 2.1 Search strategy

Two reviewers independently retrieved randomized controlled trials of alendronate for the prevention of GIOP in rheumatic diseases patients from PubMed, EMBASE, and the Cochrane Library. The search was last performed on September 7, 2015. The language of publication was not restricted. The keywords and Mesh terms used in the search included “Rheumatic Diseases” [Mesh], rheumatic diseases, rheumatoid arthritis (RA), psoriatic arthritis (PsA), systemic lupus erythematosus (SLE), ankylosing spondylitis, polymyositis, dermatomyositis, vasculitis syndrome, Still disease, polymyalgia rheumatic, systemic sclerosis, Sjogren syndrome, Behcet disease, Idiopathic Inflammatory Myopathy, inflammatory myositis, systemic vasculitis, ANCA-associated vasculitis, MCTD, UCTD, “Alendronate” [Mesh], alendronate sodium, fosamax, alendron∗, “Glucocorticoids” [Mesh], steroid∗, glucocorticoid∗, prednisolone∗, betamethasone∗, cortisone∗, dexamethasone∗, hydrocortisone∗, methylprednisolone∗, prednisone∗, triamcinolone∗, and corticosteroid∗. The Boolean operators “AND” and “OR” were used to connect these terms. The bibliographies of all included studies and other relevant publications, including systematic reviews and meta-analyses, were traced to identify the missed relevant reports. Based on the titles and abstracts, 2 reviewers selected the potential eligible studies. And then the full text of the remaining articles was examined for eligibility.

### 2.2 Inclusion and exclusion criteria

Inclusion criteria: Participants—Participants, who had a rheumatic disease, were either starting glucocorticoid treatment or had begun glucocorticoid treatment within the previous 12 weeks at any dosage of prednisone or its equivalent, and had a normal or osteopenic mean lumbar spine (LS) BMD (T-score > −2.5)^[10]^ were included. Intervention and comparison—We included following pairs of intervention and comparison. First, the intervention group was alendronate alone and the comparison group was placebo alone; second, the intervention group was alendronate along with calcium and the comparison group was calcium; third, the intervention group was alendronate along with vitamin D and the comparison group was vitamin D; fourth, the intervention group was alendronate along with calcium and vitamin D and the comparison group was calcium and vitamin D. As the effects of increasing calcium and vitamin D intake on BMD are small and nonprogressive,^[11,12]^ we performed this meta-analysis based on alendronate without consideration of calcium and vitamin D. Outcomes—The percent change in BMD from the baseline, vertebral fractures, nonvertebral fractures, and adverse events was collected as the outcomes. For publications reporting data on the same studies, we considered them comprehensively as a single study. Study—Only randomized controlled trials were included in this study.

Exclusion criteria: Participants, who exhibited metabolic bone diseases, and treatment with other drugs that might affect bone metabolism within the past 6 months, such as hormone-replacement agents, calcitonin, fluoride, phenytoin, methotrexate, cyclosporine, and oral contraceptives.

### 2.3 Data extraction and outcome measures

Two independent reviewers selected the eligible studies and extracted the following data from the included publication: the first author, year of publication, geographical location, number of patients, intervention and comparison, duration of the treatment, follow-up, patient characteristics, and study type. We contacted the first or the corresponding author for detailed study information. Any discrepancies between the 2 reviewers were resolved by an additional investigator.

The primary outcomes were the percent change in the BMD at the LS, femoral neck (FN), total hip (TH), trochanter (TR), total body (TB), vertebral fractures, and nonvertebral fractures. The secondary outcomes were adverse events, serious adverse events, and gastrointestinal adverse events. We chose the longest time point as the measurement time point.

Per-protocol data were used to analyze the percent change in the BMD from baseline whenever possible. Intention-to-treat data were used in the other variables. When standard deviations (SD) were not available in a study, standard error of the mean (SEM) was transferred into SD. If necessary, the means, SD, or SEM were extrapolated from the available diagrams and tables.

### 2.4 Risk of bias assessment

The risk of bias tool was used to estimate the quality of the included studies in accord with the Cochrane Handbook for Systematic Reviews of Interventions (version 5.1.0),^[13]^ using Review Manager, version 5.3 (The Nordic Cochrane Centre, The Cochrane Collaboration, Copenhagen, 2014). The tool has 7 fields, which included sequence generation, allocation concealment, blinding of participants and personnel, blinding of outcome assessment, incomplete outcome data, selective outcome reporting, and other biases (baseline balance and fund). A low risk of bias, a high risk of bias, or an unclear risk of bias was judged for each domain. Studies with a high risk of bias in 1 or more key items were regarded to be at a high risk of bias. Studies with a low risk of bias in all key items were regarded to be at a low risk of bias. Otherwise, they were regarded to be at an unclear risk of bias.^[14]^ Two authors independently assessed the quality of the studies, and disagreements were resolved via a discussion with a third author.

### 2.5 Quality of evidence assessment

The Grading of Recommendations, Assessment, Development, and Evaluation (GRADE) approach^[15]^ was used to grade the quality of the evidence, using GRADE Pro, version 3.6. The tool included 5 domains, which were risk of bias, inconsistency, indirectness, imprecision, and publication bias. The quality of the evidence was rated as high, moderate, low, or very low. Two reviewers independently evaluated the quality of the evidence and any disagreements were solved by discussion and consensus.

### 2.6 Statistical analysis

The meta-analysis was performed on the eligible data using Review Manager, version 5.3 (The Nordic Cochrane Centre, The Cochrane Collaboration, Copenhagen, 2014) and Stata, version 12.0 (Stata Corp, College Station, TX). The risk ratio (RR) was calculated for the dichotomous outcomes, and the mean difference (MD) was calculated for the continuous outcomes. As the clinical heterogeneity could not be excluded, we used the random-effect model^[16]^ to assess effect estimates for each outcome with an associated 95% confidence interval (CI). The I^2^ statistic^[17]^ was used to test the heterogeneity between studies. Heterogeneity was considered statistically significant if the I^2^ value was >50%. Subgroup analysis was performed to identify whether different type of rheumatic diseases (rheumatic arthritis, SLE, or other rheumatic diseases), the dose of alendronate (5 mg/d, 10 mg/d, 70 mg once/wk) affected the efficacy of alendronate. To assess the reliability of the results, a sensitivity analysis was performed by sequentially removing individual studies and recalculating the results. *P* < 0.05 was considered statistically significant and reported as a 2-sided test. Egger linear regression test and funnel plots would be implemented to estimate the publication bias.

### 2.7 Ethical statement

As all analyses were grounded on previously published studies, ethical approval was not necessary.

## 3 Results

### 3.1 Study search

**Figure 1:**
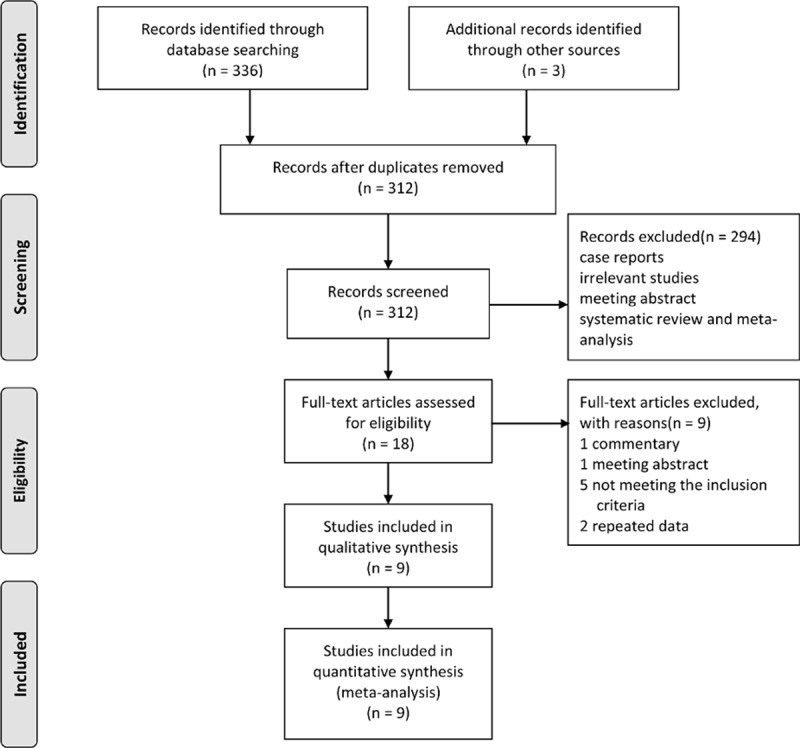
Flowchart of study selection.

Of 339 initial studies, 27 were discarded due to duplicate reports and 294 were excluded at the title or abstract level. Another 9 studies did not fulfill the inclusion criteria and were therefore excluded. Finally, a total of 9 randomized controlled trials^[7,8,18–24]^ were included in our meta-analysis. The literature screen, research selection, and reasons for exclusion were demonstrated in the flowchart (Fig. 1).

### 3.2 Study characteristics

There were 9 studies included in this meta-analysis. These trials were published between 2001 and 2009. The number of study patients in the alendronate group and control group ranged from 17 to 114 (total = 610) and 16 to 101 (total = 524), respectively. One trial was published in Chinese, and the other 8 trials were in English. When the studies were separated into individual treatment groups, there were 10 individual treatment arms compared with the controls. BMD of the LS, FN, TH, TR, and TB was measured with the same dual-energy X-ray absorptiometry (DEXA) method at baseline and the last follow-up in the different studies. Although different machines were used, such as Hologic machines (Hologic, Waltham, Mass, USA) or Lunar machines (General Electric, Madison, Wis, USA), this did not affect the measurement of BMD. The baseline characteristics of these studies were outlined in Table 1.

**Table 1:**
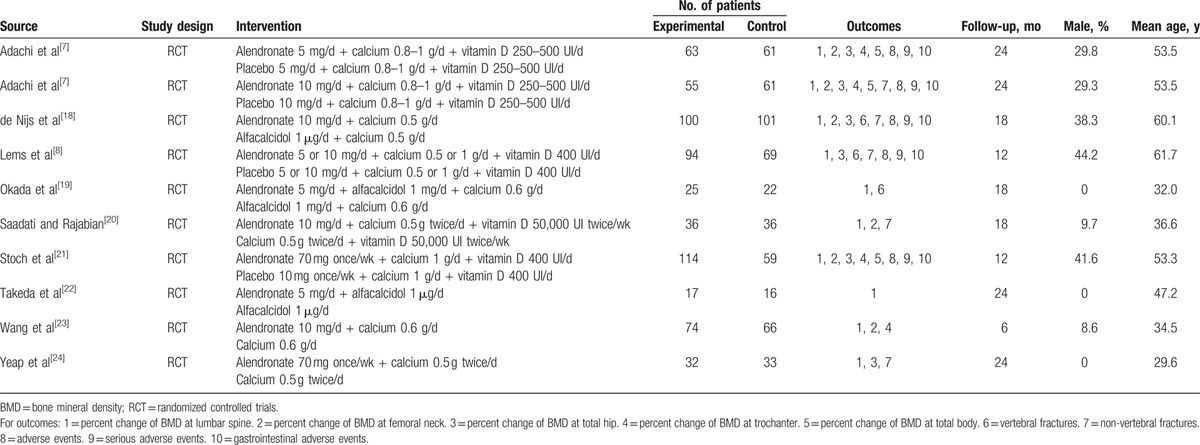
Baseline characteristics of all studies included in the meta-analysis.

### 3.3 Risk of bias in the included studies

**Figure 2:**
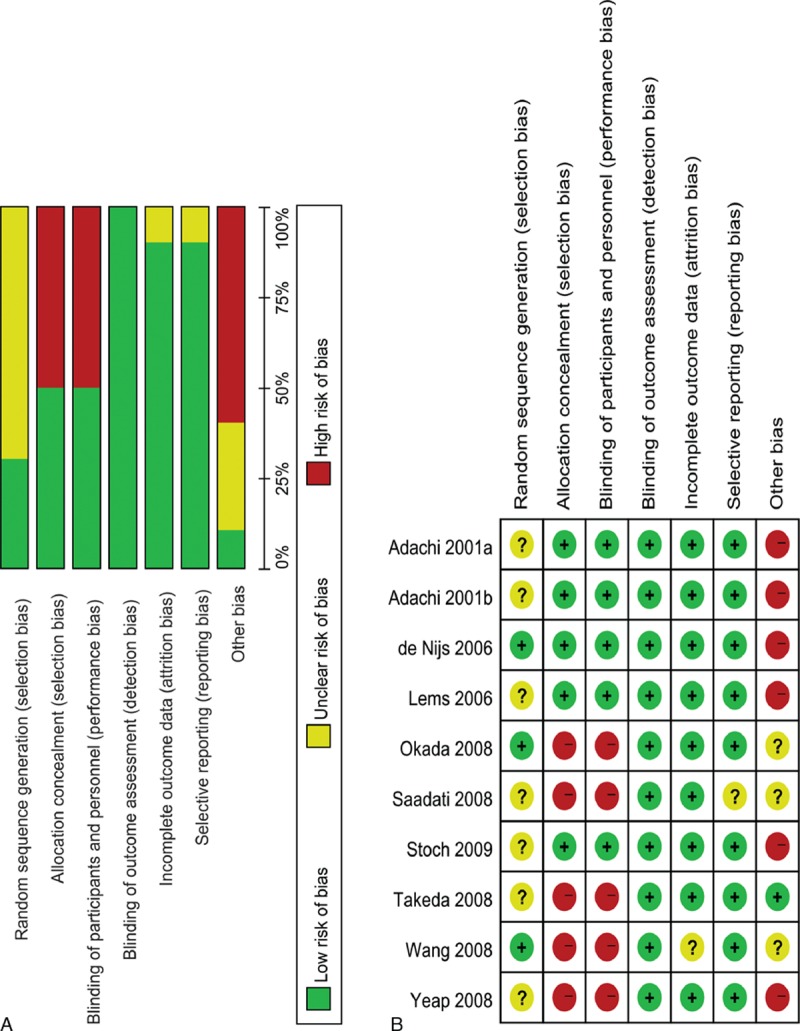
Risk of bias assessment of each included study. (A) Risk of bias graph. (B) Risk of bias summary.

The quality of included studies and the potential sources of bias were outlined in Fig. 2. All trials were judged to be at a high risk of bias. All studies reported randomization; however, only 3^[18,19,23]^ reported an appropriate random sequence generation procedure and 4^[7,8,18,21]^ described adequate concealment. Due to 5 studies^[19,20,22–24]^ performed with open-label method, blinding of the participants and personnel was not possible. All studies reported the blinding of the outcome assessors. Five studies^[7,8,18,21,24]^ received grants from industry or other types of for-profit support.

### 3.4 Percent change in the BMD at the LS, FN, TH, TR, and TB

**Figure 3:**
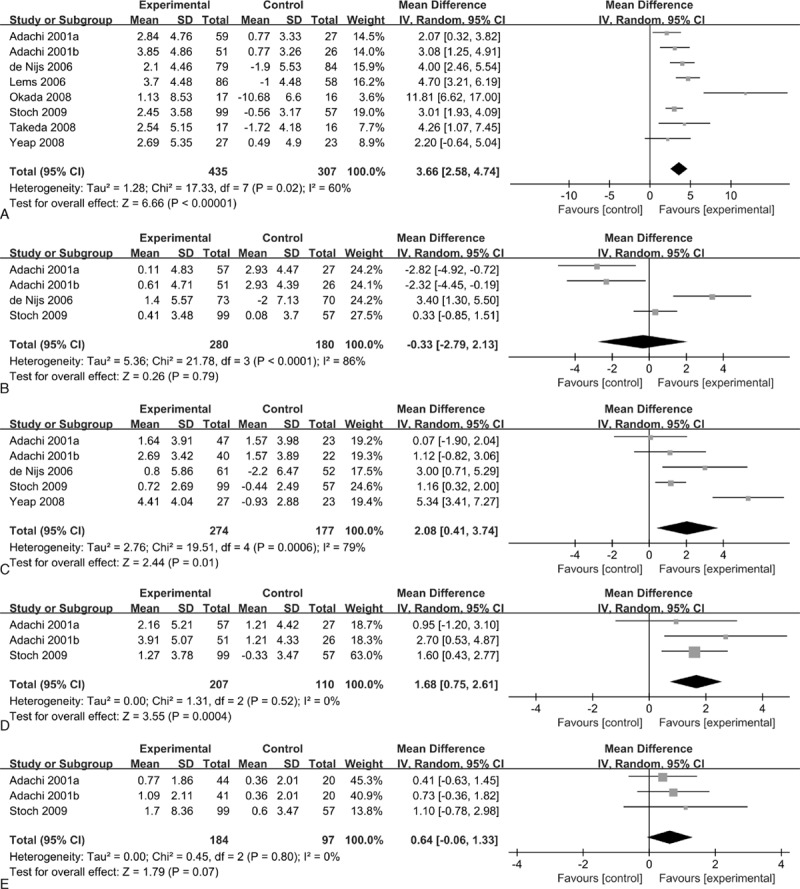
Forest plots of randomized controlled trials of alendronate in improving the percent change in the BMD at the lumbar spine (A), femoral neck (B), total hip (C), trochanter (D), and total body (E). BMD = bone mineral density.

Seven studies, including 922 patients, provided data for the percent change in the BMD at the LS. The alendronate group was associated with a significant increase in the percent change in the BMD at the LS compared with the controls (MD = 3.66, 95% CI: 2.58–4.74, *P* < 0.05; I^2^ = 60%) (Fig. 3A). The GRADE quality of the evidence was low (Table 2).

**Table 2:**
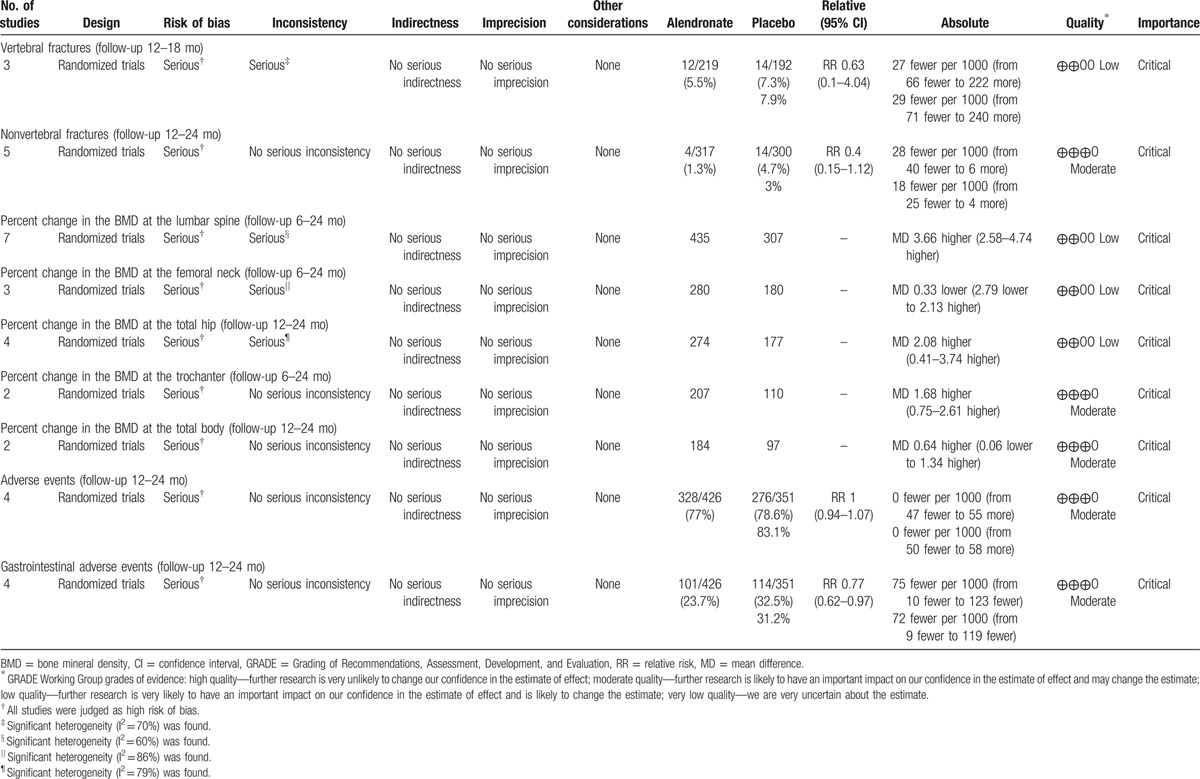
GRADE evidence profile.

The data on the percent change in the BMD at the FN were available from 3 studies (n = 614). Alendronate yielded similar results compared to the control (MD = −0.33, 95% CI: −2.79 to 2.13, *P* = 0.79; I^2^ = 86%) (Fig. 3B). The overall GRADE quality of evidence was low (Table 2).

Four studies (n = 679) contributed to the analysis of the percent change in the BMD at the TH. The percent change in the BMD at the TH was significantly increased in the alendronate group compared with the controls (MD = 2.08, 95% CI: 0.41–3.74, *P* < 0.05; I^2^ = 79%) (Fig. 3C). The overall GRADE quality of the evidence was low (Table 2).

The data on the percent change in the BMD at the TR were available in 2 studies (n = 413). There were no significant differences in the percent change in the BMD at the TR between the alendronate and control groups (MD = 1.68, 95% CI: 0.75–2.61, *P* < 0.05; I^2^ = 0%) (Fig. 3D). The GRADE quality of the evidence was moderate (Table 2).

In 2 studies (n = 413), the patients provided the data for the percent change in the BMD at the TB. We found no significant differences in the percent change in the BMD at the TB between the alendronate and control groups (MD = 0.64, 95% CI: −0.06 to 1.34, *P* = 0.07; I^2^ = 0%) (Fig. 3E). The overall GRADE quality of the evidence was moderate (Table 2).

### 3.5 Vertebral fractures

**Figure 4:**
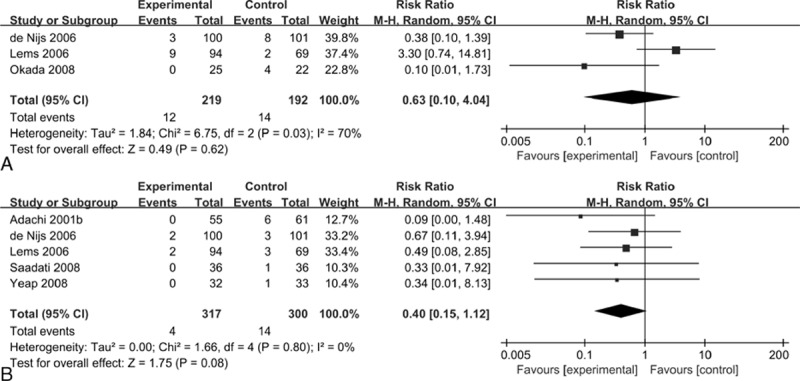
Forest plots of randomized controlled trials of alendronate in preventing vertebral fractures (A) and nonvertebral fractures (B).

Three studies (n = 411) contributed to the analysis of vertebral fractures. We observed similar rates of vertebral fractures when comparing the alendronate group with the control group (RR = 0.63, 95% CI: 0.10–4.04, *P* = 0.62; I^2^ = 70%) (Fig. 4A). The GRADE quality of evidence was low (Table 2).

### 3.6 Nonvertebral fractures

Five studies (n = 617) reported the number of patients with nonvertebral fractures. The results in patients treated with alendronate were comparable to those in the controls (RR = 0.40, 95% CI: 0.15–1.12, *P* = 0.08; I^2^ = 0%) (Fig. 4B). The overall GRADE quality of the evidence was moderate (Table 2).

### 3.7 Adverse events

**Figure 5:**
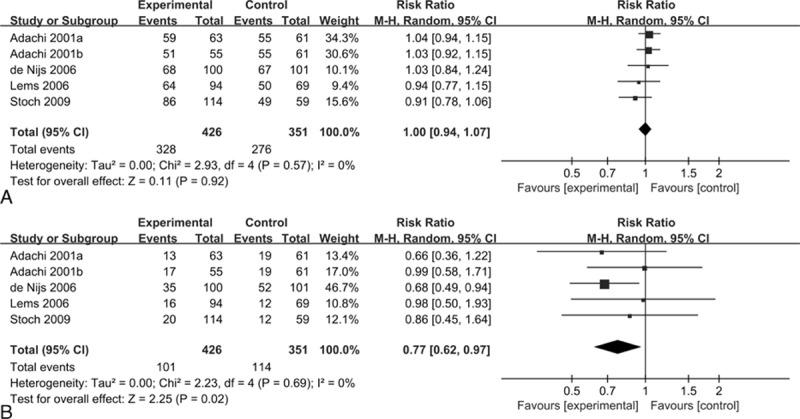
Forest plots of randomized controlled trials of alendronate in reducing adverse events (A) and gastrointestinal adverse events (B).

Four studies (n = 777) reported the incidence of adverse events. No significant differences were observed in the alendronate and control groups (RR = 1.00, 95% CI: 0.94–1.07, *P* = 0.92; I^2^ = 0%) (Fig. 5A). The GRADE quality of the evidence was moderate (Table 2). Conversely, there were significant differences with regard to gastrointestinal adverse events when comparing the alendronate group with the controls (RR = 0.77, 95% CI: 0.62–0.97, *P* < 0.05; I^2^ = 0%) (Fig. 5B). The overall GRADE quality of the evidence was moderate (Table 2).

### 3.8 Subgroup analysis, sensitivity analysis, and publication bias

**Figure 6:**
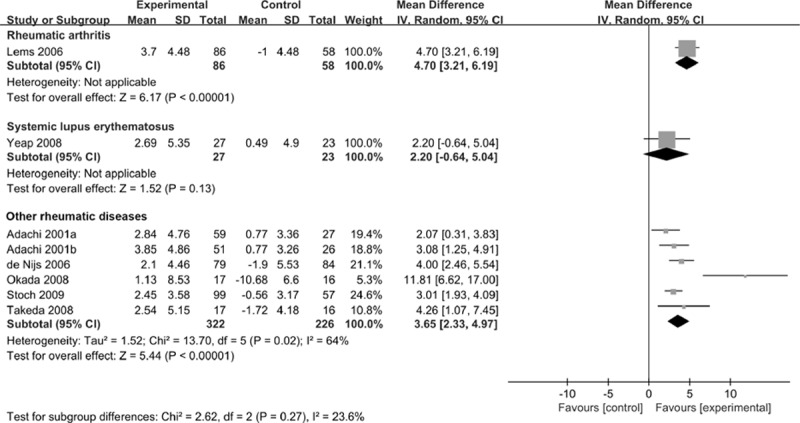
Forest plot of the percentage change in the BMD at the lumbar spine by subgroup analysis of rheumatic arthritis versus systemic lupus erythematosus versus other rheumatic diseases. BMD = bone mineral density.

**Figure 7:**
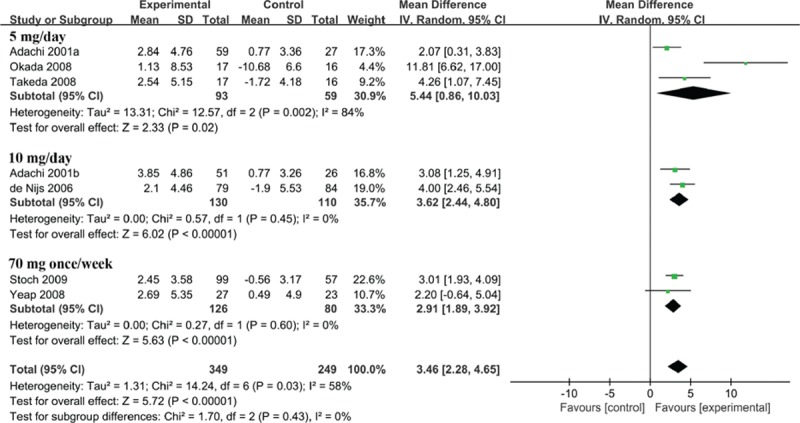
Forest plot of the percentage change in the BMD at the lumbar spine by subgroup analysis of the dose of alendronate (5 mg/d, 10 mg/d, 70 mg once/wk). BMD = bone mineral density.

Subgroup analysis was performed for the percent change in the BMD at the LS. It demonstrated that alendronate was significantly more effective than control in patients with rheumatic arthritis and other rheumatic diseases rather than SLE (Fig. 6). No matter which dose of alendronate was given to patients, alendronate significantly increased the percent change in the BMD at the LS compared with the controls (Fig. 7).

We performed sensitivity analyses by excluding each study to assess the stability of our findings. For vertebral fractures, nonvertebral fractures, the percent change in the BMD at the LS, FN, TH, TR, and TB, the pooled estimate of the remaining studies remained similar (see Supplementary Table S1).

**Figure 8:**
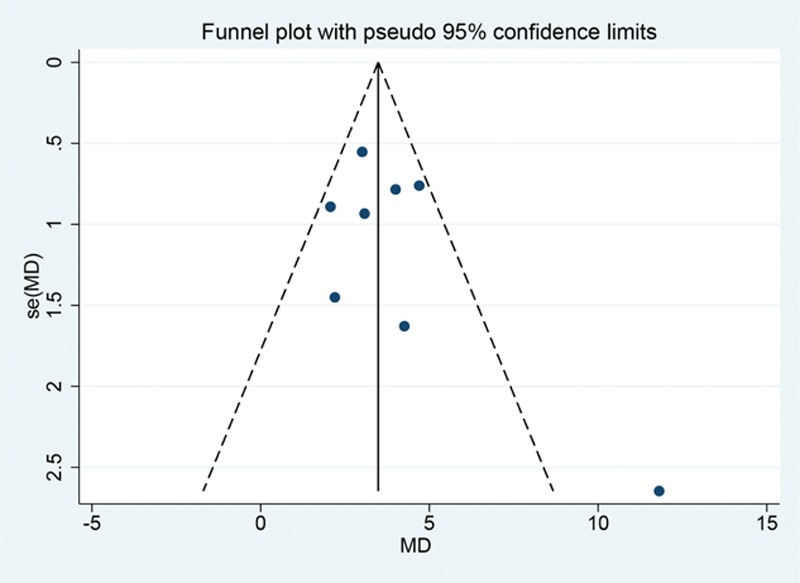
Funnel plot of the percent change in the BMD at the lumbar spine of the included studies comparing alendronate with controls. BMD = bone mineral density, MD = mean difference.

The Egger linear regression test and funnel plots were applied for the percent change in the BMD at the LS. The funnel plot was visually reviewed and did reveal some asymmetry; however, no statistical evidence of publication bias was obtained by the Egger linear regression test (*P* = 0.24, Fig. 8).

## 4 Discussion

In this meta-analysis, we evaluated the efficacy and safety of alendronate in preventing GIOP in patients with rheumatic diseases. To our knowledge, it is the first report that concentrates on preventing GIOP in patients with rheumatic diseases. By pooling the most recent evidence from randomized controlled trials, this meta-analysis comprised the largest databank of prophylaxis for GIOP in patients with rheumatic diseases.

Based on the pooled estimates, we found that alendronate increases the percent change in the BMD at the LS, TH, and TR; however, the percent change in the BMD at the FN and TB was similar in both the alendronate and control groups. For vertebral fractures and nonvertebral fractures, alendronate was not more effective than the control. Participants in the alendronate trials showed a significant reduction in gastrointestinal adverse events. In contrast, patients enrolled in the alendronate trials did not have a reduced risk of adverse events and serious adverse events.

Alendronate inhibits the enzyme farnesyl pyrophosphate synthase, thereby disrupting the production of isoprenoid lipids in the mevalonate pathway, preventing the prenylation of small GTPase proteins necessary for osteoclast function, which accounts for the antiresorptive effects of alendronate on osteoclasts.^[25]^ Our meta-analysis was similar with that of Feng et al^[26]^ in maintaining the LS and TH BMD. However, Feng et al focused on bisphosphonates, while we only investigate alendronate as a prophylactic measure for GIOP in patients with rheumatic diseases. In a meta-analysis, Yang et al^[9]^ revealed that alendronate significantly increased the BMD at the LS, with an MD of 3.91% (95% CI: 2.37–5.45) for participants with rheumatic diseases. However, the effect size was 3.66% in our meta-analysis. This may be because the alendronate treatment in the previous study played a role in prophylaxis and treatment, while it worked as a prophylactic measure for GIOP in our current study. Subgroup analysis demonstrated that alendronate could increase the percent change in the BMD at the LS in patients with rheumatic arthritis rather than SLE. However, due to the number of the included studies is small, the results are not robust. More large-scale trials are needed to evaluate the efficacy of alendronate for patients with rheumatic arthritis and SLE.

There was not a statistically significant difference in the FN BMD between the alendronate and control groups in our present meta-analysis. A previous meta-analysis^[5]^ demonstrated that there was a small statistically significant treatment effect of bisphosphonates on femoral BMD. However, it investigated the efficacy of bisphosphonates. Based on the confounding factors of several bisphosphonates, it could not be interpreted whether alendronate was bound to have a significant influence on the femoral BMD. In any event, more large-scale trials are required to assess the effect of alendronate in increasing the femoral BMD.

Alendronate is significantly important in protecting against osteoporotic fractures in postmenopausal women.^[27]^ In this study, however, we did not find that alendronate can decrease the incidence of vertebral fractures and nonvertebral fractures in rheumatic diseases. The statistical power may not be adequate in clinical trials with 1 or 2 years of follow-up; therefore, a significant difference may not be detected. However, in a previous meta-analysis, Feng et al^[26]^ found that bisphosphonates can reduce the risk of vertebral fractures in patients with rheumatic diseases, and indicated that bisphosphonates would not prevent vertebral fractures in the short term. The difference in preventing vertebral fractures may result from the pooled estimates of heterogeneous bisphosphonates. In addition, in a meta-analysis, Yang et al^[9]^ revealed that alendronate did not significantly reduce the incidence of vertebral fractures. Because fractures occurred after the threshold of osteoporosis, it was possible that significant differences did not appear in the clinical trials with a 1 year follow-up. The efficacy of alendronate in preventing vertebral fractures should be identified in studies with a longer follow-up.

The nonvertebral fractures were not reduced in participants treated with alendronate. This outcome confirmed the results of Yang et al^[9]^ and Feng et al.^[26]^ Patients treated with alendronate suffered wrist and phalangeal bones fractures, while the control patients suffered hip and tibia fractures. As alendronate increased the BMD at the TH, this may be the reason for this phenomenon. The use of alendronate as a prophylaxis for nonvertebral fractures may be clarified in a long-term trial.

The most frequent adverse events following alendronate treatment were gastrointestinal adverse events (stomach pain, nausea, gastrointestinal upset, and reflux).^[28]^ Our meta-analysis revealed that there were not significant differences in the adverse events experienced by the alendronate and control groups, and alendronate was not associated with an increased incidence of gastrointestinal adverse events. Either esophageal perforation or osteonecrosis of the jaw was a potential serious adverse event of alendronate therapy.^[29,30]^ Although these serious adverse events were rare, they should trigger a physician's vigilance. For the patients with long-term glucocorticoid treatment, the prevention of GIOP is a long-term process and attention should be paid to the adverse events of alendronate.

A major strength of this meta-analysis is that the best practice methods recommended by the Cochrane Collaboration^[13]^ were used in the present meta-analysis. This made the meta-analysis based on exhaustive literature search, sound statistical analysis method, and vividly presenting the outcome. Two assessors independently conducted the risk of bias assessments and evaluated the quality of the evidence using the GRADE approach. With the large number of studies and patients, together with the aforementioned factors, we tried to give confidence in the effect estimates on the present evidence. Moreover, a previous meta-analysis^[9]^ did not separate the prevention and treatment function of alendronate for GIOP in patients with rheumatic diseases; however, our meta-analysis was the first review to report alendronate as a prophylactic measure for GIOP in patients with rheumatic diseases, which decreased the confounding effects of different bisphosphonates and different diseases.

Our meta-analysis has limitations. First, the BMD tended to be influenced by multiple factors. Meanwhile, the diseases requiring treatment with glucocorticoids may also affect the bone quality. For example, patients with RA are susceptible to osteoporosis.^[31]^ Second, the sample sizes of some of the included studies were relatively small, and could bias the outcome. Furthermore, there were some participants who had diseases other than rheumatic diseases, and we could not exclude the data of these patients. Although the number of these patients was small, the data might affect out estimates. Moreover, there was heterogeneity among studies included in this study, and the reason may be that some studies enrolled participants with different kinds of rheumatic diseases which had diverse influences on BMD. Finally, given that the quality of some trials may be low, the authenticity of the outcome would likely be influenced.

## 5 Conclusion

Based on the current evidence, alendronate is an effective agent in preventing GIOP in patients with rheumatic diseases. Alendronate increases the BMD at the LS, TH, and TR, and is not associated with an increased risk of gastrointestinal adverse events. However, no robust evidence suggests that alendronate could protect the BMD at the FN and TB or reduce the risk of vertebral and nonvertebral fractures. With respect to the use of alendronate as a prophylactic measure for GIOP in patients with rheumatic diseases, additional large-scale randomized controlled trials with a long period of follow-up should focus on 1 type of rheumatic diseases.

## Supplementary Material

Supplemental Digital Content
