# Supplementary material for: Alendronate prevents glucocorticoid-induced osteoporosis in patients with rheumatic diseases: A meta-analysis
Source: Medicine (Baltimore). 2016 Jun 24;95(25):e3990. doi: 10.1097/MD.0000000000003990 (PMC4998340; doi:10.1097/MD.0000000000003990)
Supplement: Supplemental Digital Content [file medi-95-e3990-s001.doc]

Supplementary Table S1. Sensitivity Analysis.

|  | **Percent change in BMD at LS** | | |  | **Percent change in BMD at FN** | | |  | **Percent change in BMD at TH** | | | | | | |
| --- | --- | --- | --- | --- | --- | --- | --- | --- | --- | --- | --- | --- | --- | --- | --- |
| **Sensitivity Analysis** | **MD (95%CI)** | **I^2^ (%)** | **P** |  | **MD (95%CI)** | **I^2^ (%)** | **P** |  | **MD (95%CI)** | | | **I^2^ (%)** | | **P** | |
| Primary results | 3.66 (2.58, 4.74) | 60 | <0.00001 |  | -0.33 (-2.79, 2.13) | 86 | 0.79 |  | 2.08 (0.41, 3.74) | | | 79 | | 0.01 | |
| Omitting item |  |  |  |  |  |  |  |  |  | | |  | |  | |
| Adachi 2001a | 3.93 (2.77, 5.09) | 58 | <0.00001 |  | 0.47 (-2.26, 3.20) | 86 | 0.74 |  | 2.56 (0.60, 4.53) | | | 82 | | 0.01 | |
| Adachi 2001b | 3.79 (2.54, 5.05) | 65 | <0.00001 |  | 0.30 (-2.66, 3.27) | 88 | 0.84 |  | 2.33 (0.22, 4.43) | | | 84 | | 0.03 | |
| de Nijs 2006 | 3.64 (2.36, 4.92) | 64 | <0.00001 |  | -1.46 (-3.64, 0.73) | 78 | 0.19 |  | 1.89 (-0.07, 3.85) | | | 83 | | 0.06 | |
| Lems 2006 | 3.46 (2.28, 4.65) | 58 | <0.00001 |  | – | – | – |  | – | | | – | | – | |
| Okada 2008 | 3.37 (2.66, 4.08) | 18 | <0.00001 |  | – | – | – |  | – | | | – | | – | |
| Stoch 2009 | 3.87 (2.52, 5.21) | 63 | <0.00001 |  | -0.58 (-4.50, 3.34) | 90 | 0.77 |  | 2.38 (0.02, 4.73) | | | 82 | | 0.05 | |
| Takeda 2008 | 3.63 (2.46, 4.79) | 65 | <0.00001 |  | – | – | – |  | – | | | – | | – | |
| Yeap 2008 | 3.82 (2.66, 4.98) | 64 | <0.00001 |  | – | – | – |  | 1.20 (0.36, 2.05) | | | 18 | | 0.005 | |
|  | **Percent change in BMD at TR** | | |  | **Percent change in BMD at TB** | | |  | **Vertebral fractures** | | | | | | |
| **Sensitivity Analysis** | **MD (95%CI)** | **I^2^ (%)** | **P** |  | **MD (95%CI)** | **I^2^ (%)** | **P** |  | **RR (95%CI)** | | | **I^2^ (%)** | | **P** | |
| Primary results | 1.68 (0.75, 2.61) | 0 | 0.0004 |  | 0.64 (-0.06, 1.34) | 0 | 0.07 |  | | 0.63 (0.10, 4.04) | 70 | | 0.62 | |  |
| Omitting item |  |  |  |  |  |  |  |  |  | | |  | |  | |
| Adachi 2001a | 1.85 (0.82, 2.88) | 0 | 0.0004 |  | 0.82 (-0.12, 1.77) | 0 | 0.09 |  | – | | | – | | – | |
| Adachi 2001b | 1.45 (0.42, 2.48) | 0 | 0.006 |  | 0.57 (-0.34, 1.48) | 0 | 0.22 |  | – | | | – | | – | |
| de Nijs 2006 | – | – | – |  | – | – | – |  | 0.70 (0.02, 23.21) | | | 79 | | 0.84 | |
| Lems 2006 | – | – | – |  | – | – | – |  | 0.30 (0.09, 0.98) | | | 0 | | 0.05 | |
| Okada 2008 | – | – | – |  | – | – | – |  | 1.08 (0.13, 9.07) | | | 78 | | 0.94 | |
| Stoch 2009 | 1.82 (0.10, 3.53) | 21 | 0.04 |  | 0.56 (-0.19, 1.31) | 0 | 0.14 |  | – | | | – | | – | |

|  | **Non-vertebral fractures** | | |  |  | | |  |  | | |
| --- | --- | --- | --- | --- | --- | --- | --- | --- | --- | --- | --- |
| **Sensitivity Analysis** | **RR (95%CI)** | **I^2^(%)** | **P** |  |  |  |  |  |  |  |  |
| Primary results | 0.40 (0.15, 1.12) | 0 | 0.08 |  |  |  |  |  |  |  |  |
| Omitting item |  |  |  |  |  |  |  |  |  |  |  |
| Adachi 2001b | 0.51 (0.17, 1.51) | 0 | 0.22 |  |  |  |  |  |  |  |  |
| de Nijs 2006 | 0.31 (0.09, 1.09) | 0 | 0.07 |  |  |  |  |  |  |  |  |
| Lems 2006 | 0.37 (0.11, 1.28) | 0 | 0.11 |  |  |  |  |  |  |  |  |
| Saadati 2008 | 0.41 (0.14, 1.21) | 0 | 0.11 |  |  |  |  |  |  |  |  |
| Yeap 2008 | 0.41 (0.14, 1.20) | 0 | 0.11 |  |  |  |  |  |  |  |  |

BMD=bone mineral density. LS=lumbar spine. FN=femoral neck. TH=total hip. TR=trochanter. TB=total body. MD=mean difference. CI=confidence interval. RR=risk ratio.
